# Exploring β-catenin and TCF4 interaction in complex environments by means of novel biosensing platform focal molography

**DOI:** 10.1371/journal.pone.0333554

**Published:** 2025-09-30

**Authors:** Philipp Cedro, Roman Popov, Maxime Karrer, Jean-Christophe Hau, Eric-André Kusznir, Ralf Thoma, Andreas Frutiger, Matthias Lauer, Sylwia Huber

**Affiliations:** 1 Roche Pharma Research and Early Development, Therapeutic Modalities, Roche Innovation Center Basel, F. Hoffmann-La Roche Ltd, Basel, Switzerland; 2 Lino Biotech Ltd., Adliswil, Switzerland; 3 Roche Pharma Research and Early Development, DTA Oncology, Roche Innovation Center Basel, F. Hoffmann-La Roche Ltd, Basel, Switzerland; Rutgers: Rutgers The State University of New Jersey, UNITED STATES OF AMERICA

## Abstract

Analyzing biomolecular interactions is essential for drug discovery, aiding the design of new candidates by revealing their action with targets. Various biophysical methods are routinely applied to characterize binding properties between molecules. Recently, focal molography, a novel sensor-based technology, has been developed to study interactions in complex environments. It measures changes in the intensity of diffracted light at a focal point due to analyte binding to a patterned array of binding sites, known as a mologram. Focal molography filters a specific binding signal from nonspecific background binding, allowing holistic analysis in biologically relevant environments. We present binding data and method’s validation in both, buffer and complex media, using focal molography and compare results to the gold standard method, surface plasmon resonance. Our model system focuses on the interaction between β-Catenin and a T-cell factor 4. β-Catenin, crucial in gene regulation for cell proliferation and differentiation, is a key target in cancer therapeutics. Confirming focal molography’s ability to accurately measure binding affinities creates a reliable methodology that fills gaps in current drug discovery techniques.

## Introduction

**Fig 1:**
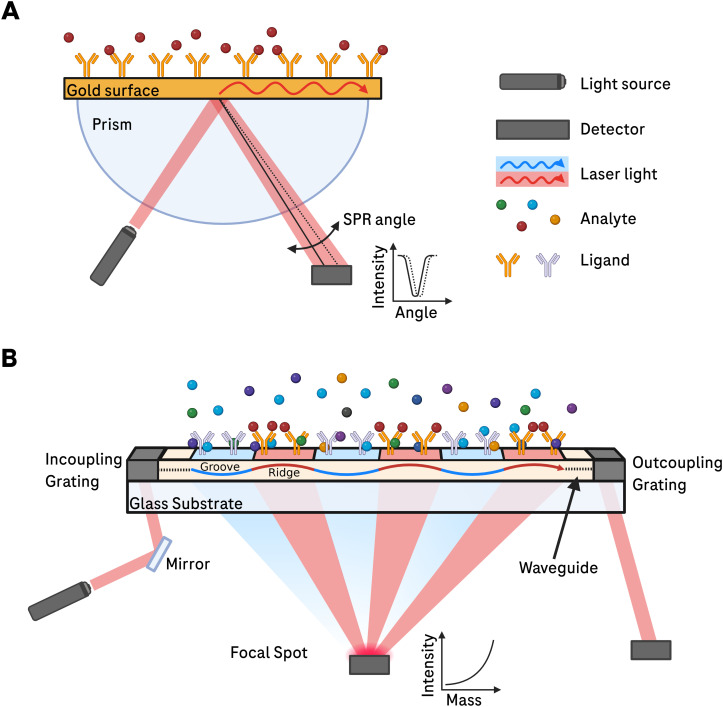
Comparison of surface architecture and signal generation of SPR versus FM. (A) In SPR, the incoming laser light excites surface plasmon polaritons at the gold liquid interface. The momentum of the incident mode needed for the excitation is dependent on the refractive index in the vicinity of the surface (in the evanescent field of the polariton). Hence, there exists an intensity minimum at a certain angle of reflection for a given wavelength, which can be detected by various interrogation schemes (angular, wavelength and intensity). Upon binding of analytes to the surface, the refractive index changes and therefore the momentum needed for the excitation of the polaritons. This leads to a change in the angle at which the minimum of intensity is recorded (angle interrogation). In an SPR experiment, the change in angle of the intensity minimum is recorded over time to create a sensogram. (B) In FM, coherent incoming laser light is coupled to the waveguide on the sensor surface at the incoupling grating. Then, the guided wave propagates in the waveguide and illuminates via the evanescent field the mologram (a coherent assembly of recognition elements called ridges and grooves). Part of the evanescent field is coherently diffracted into the focal spot by constructive interference (red shading). The intensity of this spot is quadratically proportional to the amount of bound mass of the analyte difference between ridges and grooves. Random scattering from non-specific binding at ridges and grooves destructively interferes (the phase of scattered light is shifted by half a wavelength between the center of the red and blue line) and only linearly contributes to the intensity of the focal spot (blue shading). Then, at the end of the sensor, an outcoupling beam is measured in order to translate the measured intensity into a diffraction efficiency of the mologram and from this into a coherent mass density. In a FM experiment, the change of intensity (diffraction efficiency) is recorded over time to create a sensogram. In SPR, referencing is carried out by measuring two sensors in separate channels (mm apart) independently and subtracting the reference from the signal digitally. Contrary, in FM referencing is intrinsic (due to alternating ridges and grooves, only a 200 nm apart) and is carried out by the wave nature of light. Only the difference is measured by the intensity in the focal spot. This self-referencing on the submicron scale over 1000 of signal and reference regions per spot and direct differential detection by diffraction is what renders FM much more robust than refractometric sensors (e.g., SPR). Figure was created with BioRender.

Biological systems are regulated by interactions between two or more molecules in biomolecular pathways. Those interactions are governed by the principles of thermodynamics, electrostatic forces, structural elements and their molecular recognition in a specific context [1]. To elucidate the molecular mode of action (MMoA) between interacting molecules, it is essential to comprehensively understand and characterize the processes occurring at the molecular level of the interaction [2]. In drug discovery, information that can be obtained about the MMoA between a malfunctioning biomolecule and a chemical compound is of utmost importance. The discovery of a drug that repairs a malfunctioning biological pathway is pivotal for developing treatments for the medical conditions that that arise from such dysfunctions [3]. Today, a variety of technologies are employed to characterize different types of molecular interactions [4–11]. Surface plasmon resonance (SPR) is likely the most widely used and versatile method for studying the kinetics of biomolecular interactions [12–14] and quantifying binding affinity between various molecules. For example, SPR can measure interactions between proteins, protein-small molecules, protein-nucleic acids, protein-carbohydrates, protein-lipids, and combinations thereof [15]. All these types of interactions are measured by immobilizing a ligand molecule on the SPR sensor and introducing an interaction partner (analyte) in solution to the sensor surface. Upon binding of the analyte to the ligand, the refractive index in the vicinity of the surface changes, leading to a shift in the resonance condition at which the plasmon is excited. This results in a measurable change of the reflected light as a function of incidence angle or wavelength. Consequently, the association and dissociation of the ligand-analyte complex can be monitored in real time (Fig 1A) [15].

Although new and advanced analytical tools have been developed in recent years, the majority of available methods are still limited to measurements in simplified buffer systems and rely on working with purified target molecules [16]. In contrast, focal molography (FM) proposes a novel, label-free measurement platform to quantify and characterize binding events directly in complex biological environment [16–20]. To investigate the binding parameters of two molecules in their native context, two criteria must be met [16]. Firstly, the measurement must be sensitive enough to detect small signal changes upon binding. Secondly, it must clearly distinguish specific binding to the recognition sites from non-specific binding towards the sensor surface [1,21]. In the case of the SPR technique, any binding event observed on the sensor surface, including non-specific binding, leads to a change of refractive index and thus resonance signal. Non-specific binding in SPR results from its sensor matrix complexity and charge interactions, where biomolecules and charged molecules adsorb onto the sensor. Hydrophobic interactions and protein aggregation also contribute to non-specific adsorption on the sensor surface. Those sources were discussed broadly by other investigators [22]. Therefore, in SPR assays, the resonance signal measured on the active surface (with the immobilized target ligand) must be appropriately referenced against signals collected from a reference surface lacking the targeted binding sites. Conversely, FM intrinsically references the binding signal by its sub-micron interdigitated reference subtraction mechanism. In FM, coherent laser light diffracts at coherently arranged molecules that form a focusing diffraction grating on an optical waveguide [23]. The blueprint (i.e., the underlying binding pattern) of this molecular diffraction grating is known as a mologram, originating from a combination of the words “molecule” and “hologram” [16]. The mologram consists of a repeating pattern of approximately 1000 line pairs per sensor spot, comprising high-affinity ligand molecules (referred to as ridges) and non-binding molecules (referred to as grooves). Light scattered by specifically bound molecules on the mologram interferes constructively at the mologram’s focal spot located a few millimeters beneath the sensor surface (Fig 1B). The method is fully quantitative, with the intensity at the focal spot proportional to the square of difference in the number of molecules bound to the ridges vs the grooves. Molecules that bind randomly or equally to both ridges and grooves contribute negligibly to the intensity of the focal spot. Specifically, the signal from non-specifically bound molecules is only proportional to the number of such molecules, not the square of their number. This is significant; for example, 1000 molecules coherently arranged on the ridges produce the same signal as 1 million molecules equally distributed (Poisson distributed) across ridges and grooves. This effectively eliminates non-specific binding contributions to the recorded signal in the focal spot [16,21,24]. Thanks to this unique property, binding events can be monitored in real time and label-free, even in complex media [1,16,19,21,24–27]. Achieving the same level of noise rejection with SPR would require aligning 1000 signal and reference channels and reading them out with one single measurement – an approach that is practically impossible [28].By eliminating signal changes caused by background binding events, FM enables the development of new assays to study biomolecular interactions in more complex environments [18,21,29,30]. For example, Gatterdam *et al.* [21] conducted binding analyses of the interaction between an antibody and the β-amyloid peptide which is relevant for Alzheimer’s disease research. They demonstrated that dissociation constants could be determined in both buffer and serum, achieving a low nanomolar limit of detection (LOD) [21]. This performance in buffer is comparable to the best published data from state-of-the-art label-free SPR instruments [21,31,32] and marked the first demonstration of FM’s real-time binding capabilities in both buffer and serum. Other approaches to characterize such interactions would have required extensive optimization for protein purification multiple assays [30,33]. Dirscherl *et al.* recently demonstrated FM’s ability to characterize binding interactions (K_D_, k_on_, k_off_) and compared it to SPR and bio-layer interferometry (BLI), also in context with problematic analytes that are prone to non-specific binding [20]. FM goes beyond traditional kinetic analysis in serum, Reichmuth *et al.* successfully measured interactions of membrane proteins directly from cellular surfaces *in vivo* [18,29]*.* They further extended the method to measure intracellular phosphorylation of extracellular signal-regulated kinases (ERK) in real-time, label-free [29,34]. FM has also shown possible applications in diagnostic and patient stratification by utilizing a multiplexed array of different hemagglutinin (HA) peptide variants to distinguish blood plasma from different donors [18].

In this study, we expand the application scope of FM to interaction measurements in cell lysate to investigate the protein-peptide interaction between β-catenin and T-cell factor 4 (TCF4). β-Catenin is a critical regulator of cellular development and tissue homeostasis, functioning through its interaction with T-cell factor 4 (TCF4) [35]. The β-catenin/TCF4 complex plays a pivotal role in Wnt signaling, where β-catenin accumulates in the cytoplasm upon Wnt activation and then subsequently translocates to the nucleus [36–38]. In the nucleus, β-catenin forms a complex with TCF4, a transcription factor that regulates the expression of target genes involved in cell proliferation and differentiation. The interaction between β-catenin and TCF4 is tightly regulated by various post-translational modifications [36]. Beyond its role in normal cellular processes, dysregulation of the β-catenin/TCF4 interaction has been implicated in many diseases, including cancer progression and homeostasis [39,40]. Alterations in this signaling pathway can result in uncontrolled cell growth and malignancies. Understanding the specific mechanisms governing the interaction between β-catenin and TCF4 offers valuable insights for developing therapeutic interventions targeting Wnt/β-catenin signaling in disease contexts [35]. β-catenin was selected for this FM study based on several features. The protein scaffold serves as an interaction platform for many β-catenin binding partners at the membrane, in cytosol, and in the nucleus and thus its binding behavior may differ when analyzed in complex biological environment in comparison to simplified buffer system. Furthermore, it possesses an elongated structure resulting in structural flexibility which can enhance unknown allosteric effects while present in biologically relevant environment. Considering both facts, β-catenin is a complex and challenging target for direct binding assays and meaningful biological characterization [35,41]. Using the β-catenin/TCF4 interaction as our model system [35–40], we aim to validate the FM method’s robustness. By replicating SPR data from Yu *et al.* [42], we reproduced these interactions in both buffer solutions and cell lysates with FM. This provides comprehensive affinity analyses, extends findings by Yu *et al.* [42], and addresses SPR limitations. FM’s self-referencing in near-physiological conditions like cell lysates could enhance its application in drug discovery. Here, we present data demonstrating the versatility of FM technology to characterize biomolecular interactions in both simple and complex environments. Specifically, we demonstrate: (i) the transferability and adaptation of binding assays previously established on SPR sensors to FM sensors; (ii) reproducibility of binding data monitored in buffer and cell lysate; (iii) the comparability of both methods when applied in a buffer system; (iv) FM’s ability to detect specific interactions in cell lysate; (v) method’s reproducibility and thus robustness of the FM technology.

## Materials and methods

### SPR experimental setup

For all SPR experiments, the running buffer consisting of 10 mM HEPES, 150 mM NaCl, 0.01% (v/v) P20, 1% (v/v) glycerol, 0.01% (v/v) BSA and 2% (v/v) DMSO at pH 7.4 was used. The TCF(7–30) peptide (GGGDDLGANDELISFKDEGEQEEK-K-biotin-amide) peptide was obtained from Biosyntan GmbH. Experiments were conducted at room temperature on a Biacore T200 (GE Healthcare, Uppsala, Sweden). The peptide was immobilized onto a Series S sensor chip SA (10352113, Cytiva, Uppsala, Sweden). Following the manufacturer’s protocol, the sensor was first prepared, and the peptide was injected at a concentration of 10 nM at a flow rate of 10 µL/min. Immobilization was achieved via biotin-streptavidin interaction to a level of 5, 25 and 50 RU in three different flow channels respectively. Finally, all flow channels including the reference channel were passivated with free biotin at 500 nM. Binding analysis was monitored on all four flow channels with sample association time of one minute and dissociation time of four minutes at a flow rate of 50 µL/min. Regeneration (i.e., removal of β-catenin from ligand) of the sensor surface was achieved by injecting 50 mM glycine (pH 9.5) for three minutes at a flow rate of 100 µL/min. Even though this step was not necessary for SPR, it was implemented in order to match the assay setup from FM.

### FM experimental setup

FM experiments were conducted using a prototype FM reader from the “Fermi” generation developed by lino Biotech AG (Supplementary Figure 10 S1 File). The flow channel shape was hexagonal (Supplementary Figure 9 A&C S1 File) and the parallel region measured 12.0 x 1.9 x 0.1 mm. Only 18 molograms were recorded per measurement in order to save computing power for faster sampling rates of the intensities of the single molograms. Thus, the lower part of the double flow chamber was used. In all FM experiments, the same running buffer as in SPR was used. Samples in glass vials were injected from the autosampler (Alias, Spark Holland) and transferred with a syringe pump (Precision Syringe Drive/4, Hamilton) to the flow channel for 17 seconds at a flow rate of 400 µL/min (sample delivery step). This purged the chamber and reduced the influence of concentration gradients due to Taylor dispersion in the delivery tubing, which had an internal diameter of 250 µm and a length of 80 cm. During the association phase, the sample was passed through the flow chamber for one minute at a flow rate of 50 µL/min. To prevent the recording of the dilutive end of the sample, a fast flow rate of 500 µL/min was applied for two minutes to record the dissociation phase, followed by additional two minutes at a flow rate of 50 µL/min. For the regeneration, regeneration solution was injected from glass vials into the autosampler loop. The regeneration solution was passed through the flow chamber for three minutes at a flow rate of 100 µL/min.

### Chip fabrication

A sensor chip of FM measured 9 x 18 mm. The sensing area per mologram was 400 x 400 µm with 54 molograms (6 x 9 array) being present on one sensor chip [21]. The center to center spacing of molograms in flow direction was 690 µm and 750 µm perpendicular to the flow. The sensors used in FM were manufactured and treated following published manufacturing protocols [16,18,21] with the difference that the photoprotective PhSNPPOC group was replaced by a more hydrophilic PySNPPOC group. To activate the ridges using reactive immersion lithography, a phase mask was placed on the surface with a layer of 0.1% (v/v) hydroxyl amine in DMSO applied in between the mask and the sensor surface. The chip was exposed at 405 nm at a dose of 4000 mJ/cm^2^ with a custom-built setup [17]. Following exposure, the chip was incubated in 1 mM methyltetrazine-4-polyethyleneglycol-N-Hydroxysuccinimide (MeTz-PEG4-NHS) dissolved in HBST (10 mM HEPES, 150 mM NaCl, 0.05% (v/v) Tween20, pH 8.0) for 1 hour. After incubation, the chip was washed sequentially with 50:50 H_2_O/DMSO, DMSO, Isopropanol and H_2_O. Next, the entire chip surface, including the grooves, was activated by flood illumination with a dose of 18 J/cm^2^. The chip was washed sequentially with 50:50 MQ H_2_O/DMSO, DMSO, Isopropanol and MQ H_2_O. As a final step, the chip was incubated with 10 mM tetrazine (Tz)-PEG5-NHS in HBST (10 mM HEPES, 150 mM NaCl, 0.05% (v/v) Tween20, pH 8.0) for 1 hour and washed with isopropanol and H_2_O.

### Peptide immobilization in FM

The initial sensor surface for FM consisted of MTz chemical linkers in the ridges and Tz chemical linkers in the grooves. Trans-cyclooctene (TCO) labelled TCF4(7–30) peptides were immobilized onto the FM sensor surface via click chemistry. First, mutant TCF4(7–30) was manually pipetted (100 uL) at 10 µM (in PBS) into an open flow chamber (Supplementary Figure 9 B&C in S1 File) on the sensor. After aspirating the mutant TCF4(7–30) peptide, the binding TCF4(7–30) peptide was injected manually (100 uL) at 20 µM (in PBS) concentration. For all sensors produced for the FM experiments, an immobilization level of approximately 3 pg/mm^2^ of the TCF4(7–30) binding peptide was used.

### Proteins and peptides

Human β-catenin was procured from Proteros Biostructures (Variant: β-catenin (human) (Hs134–670)+TCF4(Hs1–53)). The β-catenin binding peptide TCF4(7–30) (TCO-PEG3-C-GGGDDLGANDELISFKDEGEQEEK), the non-binding (D16A/E17A) mutant version TCF4(7–30) (TCO-PEG3-CGGGDDLGANAALISFKDEGEQEEK) and TCF4(7–51) (TCO-PEG3-C- GGGDDLGANDELISFKDEGEQEE KSSENSSAERDLADVKSSLVNE) were obtained from LifeTein, LLC.

### Binding analysis

The equilibrium dissociation constant (K_D_) and half maximal inhibitory concentration (IC_50_) were determined from binding signals collected in direct binding and competition experiments, respectively. To achieve this, various concentrations of β-catenin and the strong binding competing peptide TCF4(7–51) [42] were injected onto the SPR and FM sensors. β-catenin was titrated up to 1000 nM (7 concentration points with a dilution factor of 2 with one blank injection run at the beginning) in direct binding experiments. In the competition experiments, the β-catenin was kept at constant concentration of 250 nM and the competing peptide TCF4(7–51) was titrated up to 1000 nM (0, 50, 100, 125, 150, 175, 200, 250, 500, and 1000 nM).

### Cell culture

RKO cells were obtained from the American Type Culture Collection (ATCC, CRL-2577). The cells were cultured in Dulbecco’s Modified Eagle Medium supplemented with 10% fetal bovine serum and 100 μg/ml penicillin-streptomycin (all from Gibco) under humidified atmosphere containing 5% CO2 at 37°C.

### Cell lysis

The pelleted cells were resuspended in PBST (10 mM phosphate buffer, 2.7 mM KCl, 137 mM NaCl and 0.05% (v/v) P20, pH 7.4) to a concentration of 20 Million cells per mL. To this suspension, cOmplete^TM^ protease inhibitor (Roche) and the detergent C12E8 were added to a final concentration of 1%. The cells were lysed for one hour at 4°C while being vertically rotated at 5 rpm. For storage, the lysate was clarified by centrifugation at 14’000 g for 15 minutes at room temperature. The supernatant was removed, and the resulting cell lysate pellet was stored at −20°C.

### Data analysis

The SPR data was visualized and analyzed using GraphPad Prism 10.0.0 (Boston, Massachusetts USA, www.graphpad.com). For SPR analysis, signals recorded on the active surfaces were referenced with signals from the reference channel. The signal responses observed in equilibrium were normalized to 100% and plotted against the concentration of protein or peptide, respectively. This allowed the calculation of K_D_ (specific binding with hill slope) and IC_50_ (log(inhibitor) vs. normalized response -- Variable slope) values using non-linear regression fit with the following formula:


$$R_{eq}=\;\frac{\text{1}\text{0}\text{0}}{{\text{1}\text{0}}^{h\times (logX-logC)}+\text{1}}$$ (1)

Where R_eq_ being the response in equilibrium, C the concentration of the injected protein (K_D_) or peptide (IC_50_), h the hill slope [43] and X being the fitted K_D_ or IC_50_, respectively. The FM data were analyzed using custom made software (lino Biotech AG). The software utilized the python lmfit library [44] to fit the binding signal in equilibrium with respect to protein and peptide concentration. The processed data was then visualized in GraphPad prism.

## Results and discussion

### Click chemistry mediated backfilling of the molosensor surface

**Fig 2:**
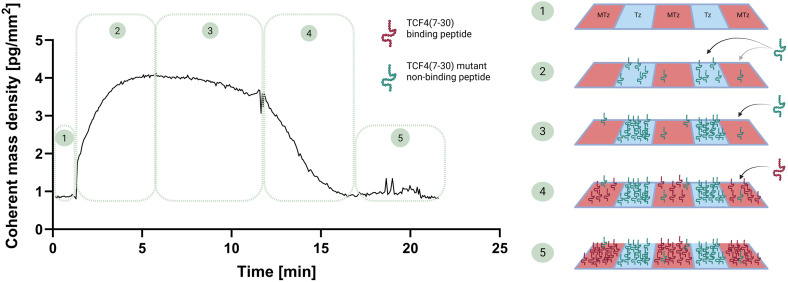
Backfilling the FM sensor surface with click chemistry. Left: The time trace sensogram collected while backfilling of FM sensor with TCF4(7-30) peptide and its mutant. The median trace out of 18 molograms is shown. The coherent mass density increase is displayed on the y-axis. The x-axis shows the time dimension. Right: Scheme of backfilling process on the sensor surface. Initial surface chemistry; MTz in ridges and Tz in grooves (Step 1). In step 2, the non-binding mutant peptide was injected and primarily reacted with Tz in grooves. In step 3, since all Tz is depleted in grooves, the peptide only immobilized in ridges. In step 4, the binding peptide was only immobilized in the ridges until the signal decreased back to baseline. Step 5 depicts fully backfilled sensor surface. Figure was created with BioRender.

For the immobilization of the TCO labelled peptides TCF4(7–30) (specific for β-catenin) as well as the mutated, non-binding version TCF4(7–30) the TCO click chemistry [45] on the sensor surface was used. Here, the sensor surface previously modified with tetrazine (Tz) in grooves and methyltetrazine (MTz) in ridges was applied to immobilize native and mutated peptide, respectively. The mologram notation follows the convention [ridges|grooves]. The response in FM has a unit of pg/mm^2^ and refers to “coherent mass density”. The word “coherent” is important as only the mass difference between ridges and grooves is detected and not the total mass density bound to the surface. The conversion factor between coherent mass density and mass density is known as analyte efficiency [19]. Using the aforementioned peptides, a backfilled (affinity matched) [18] sensor surface [TCF4(7–30)|mutated TCF4(7–30)] as described in previous studies (Fig 2) was generated. A backfilled sensor further improves correction for nonspecific binding by matching biochemical properties of ridges and grooves. This design ensured that non-specific binding towards the peptide was likely to occur uniformly and the sensor will have a flat baseline upon the injection of cell lysate. Importantly, while backfilling is not always necessary, it becomes critical for large ligands (due to their greater exposed surface area) and more crowded complex media. The non-specific binding towards the sensor surface itself is referenced out in any case. To create a backfilled sensor, the two peptides were immobilized at similarly matched ligand density on the ridges and grooves. This approach minimized the initial baseline drift, thereby enhancing sensitivity by improving the signal-to-noise (S/N) ratio, and by promoting an equal distribution of non-specific binders on ridges and grooves. Initially, the sensor surface chemistry consisted of MTz in the ridges and Tz in the grooves (Fig 2, step 1). In the first step, the non-binding TCF4(7–30) mutant was injected onto the sensor. Since the reaction with Tz is faster than with MTz [46], the peptide predominantly immobilizes in the grooves, creating a mass difference between ridges and grooves and resulting in an increase in signal (Fig 2, step 2). Once the Tz linkers in the grooves were depleted, the peptide continued immobilizing in the ridges. As a result, the coherent mass difference between ridges and grooves decreased, leading to a reduction in signal (Fig 2, step 3). In the next step, the protein-specific peptide TCF4(7–30) was injected. This peptide could only bind free MTz linkers in the ridges (Fig 2, step 4) until the sensor surface became fully saturated (Fig 2, step 5).

TCF4(7–30) was successfully immobilized with a ligand density of approximately 3 pg/mm^2^, and the sensor was backfilled with mutated TCF4(7–30) covalently on the FM sensor surface using TCO – Tz click chemistry (Fig 2). The increase of 3 pg/mm^2^ for the TCF4(7–30) peptide corresponds to a coherent ligand density of roughly 1.2 fmol/mm^2^ (using a molecular mass of 2500 g/mol for the peptide). To get the real ligand density present on the sensor from the coherent ligand density an assumption for the analyte efficiency is required, it was shown previously that the analyte efficiency is between 10–20% [17,26]. Therefore, the ligand density on the surface is around 5–10 fmol/mm2. In contrast, to immobilize TCF(7–30) on the SPR sensor, the biotin-streptavidin approach was applied. The interaction between biotinylated TCF4(7–30) and the streptavidin on the SPR sensor is highly affine, effectively mimicking covalent binding, and is routinely used as an immobilization strategy in the SPR technique [13,47–49]. Furthermore, the morphology of the two sensor architectures differed in the arrangement of the binding sites in the sensing volume. In FM, the binding sites are rather arranged in a two-dimensional, planar configuration with a layer thickness of 10–20 nm. In SPR, however, for the employed sensors the binding sites are attached via streptavidin to a dextran matrix with a layer thickness of 100 nm [12]. Thus, in the SPR experiments ligand densities approximately one order of magnitude higher can be achieved. This increases the sensitivity towards the analyte, especially for small molecule studies. While SPR currently offers an advantage in detection of small molecule and fragments due to its three-dimensional sensor surface architecture, the two-dimensional planar configuration of FM still provides robust performance for interactions where such high mass sensitivity is not required. FM can also become well-suited for a broader range of analytes including small molecules exemplifying weak or covalent binders.

### Focal molography allows quantification of protein-peptide interaction in buffer and complex media

Having established robust ligand immobilization strategies for both FM and SPR, the next step was to quantitatively characterize the binding affinity and binding specificity of the monitored β-catenin–TCF4(7–30) interaction. By comparing the equilibrium binding parameters derived from both platforms, and performing experiments in both simple buffer and complex cell lysate environments, we (i) determined the binding affinity constant (K_D_) in both buffer and cell lysate, (ii) confirmed the specificity of the observed interactions in competition experiments, and (iii) assessed the reproducibility of FM-based binding data both within and across sensors by performing three individual runs on the same sensor and over three different sensors.

**Fig 3:**
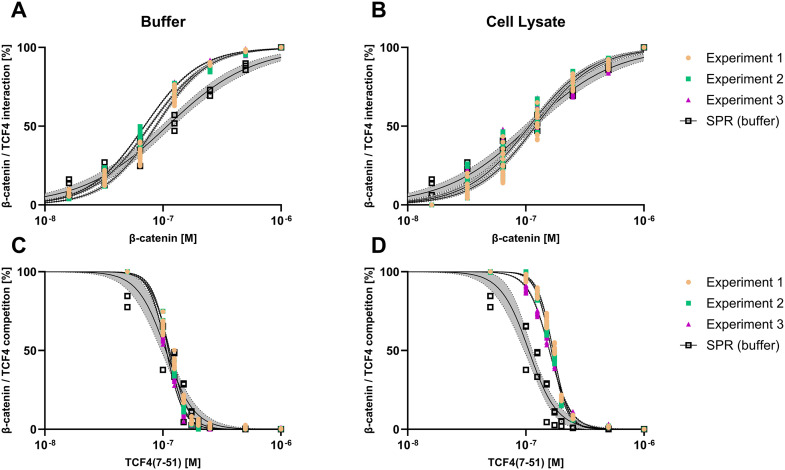
FM and SPR measurements of β-catenin/TCF4 interactions and β-catenin competition with a free peptide. Normalized sigmoidal dose response (y-axis) of β-catenin (x-axis) response versus concentration monitored in buffer (A) and cell lysate (B). Normalized sigmoidal dose response (y-axis) of TCF4(7-51) (x-axis) response in solution with β-catenin versus concentration monitored in buffer (C) and cell lysate (D). For FM experiments, a representative sensor containing 18 molograms, the normalized equilibrium response (R_eq_) for each injection of protein or peptide is plotted from 3 individual experiments. For SPR experiments, the normalized equilibrium response (R_eq_) for each injection of protein or peptide from 3 experiments on the same sensor containing 3 different immobilization densities (5, 25, 50 RU, which under the assumption 1 pg/mm^2^ = 1 RU, corresponds to 2, 10, 20 fmol/mm^2^) is shown. The 95% confidence interval band is shown as dotted line for each experiment. The normalized sigmoidal dose response curve obtained from SPR experiments in buffer are shown in all plots for comparison to FM data. The sigmoidal dose response curves were fitted with the non-linear regression model in Graphpad Prism 10.4.1 (log(inhibitor) vs. normalized response – Variable slope).

**Fig 4:**
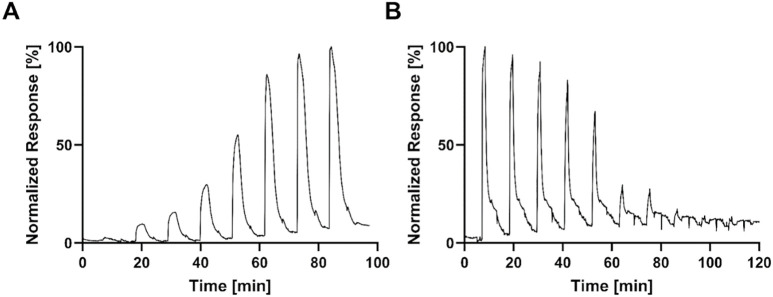
Exemplary time traces of beta-catenin titration over TCF4(7-30) peptide surface and corresponding competition experiments with a free TCF4(7-51) peptide collected with FM. (A) Dose response of beta-catenin (up to 1000 nM) to determine binding affinity constant; (B) Competition of beta-catenin by a free peptide TCF(7-51) titrated in dose response (up to 1000 nM) at 250 nM beta-catenin.

To determine the binding affinity constant (K_D_ value) binding experiments with β-catenin were performed on the SPR sensor, functionalized with biotinylated TCF4(7–30) at three different immobilization densities, as well as on the FM sensor, functionalized with TCO-TCF4(7–30) on the ridges and backfilled with mutant TCO-TCF4(7–30), in buffer (Fig 3A) and cell lysate containing 1 Mio cells/mL (Fig 3B). To assess the half maximum inhibition concentration (IC_50_), β-catenin in solution was supplemented with the free competing peptide TCF4(7–51), which targets the same binding pocket on the protein as TCF4(7–30) immobilized on the sensor surface. Experiments were conducted in both buffer (Fig 3C) and cell lysate containing 1 Mio cells/mL (Fig 3D). The concentrations of the injected protein and free peptide are listed in the method section. Time traces of all experiments are provided in the Supplementary Materials (SPR, Supplementary Figure 3; FM, Supplementary Figure 1 and 2 in S1 File) and exemplary time traces for the dose response and competition experiment are shown in Fig 4.

For the data obtained in SPR experiments in buffer (Fig 3), with non-linear regression fit (using formula (1)), a K_D_ of 96–121 nM (in the 95% confidence interval (CI)) was determined. Using formula (1) for the calculation of IC_50_, the measured values ranged from 95 to 115 nM (within the 95% CI). This data is in a good agreement with the findings presented by Yu *et al.* [42]. From the attempts of experiments with cell lysate on the SPR sensor, no information could be gathered about the interaction affinity of β-catenin towards the TCF4(7–30) immobilized on the surface due to massive, non-specific background interactions (Supplementary Figure 6 S1 File). In contrast, when injecting the RKO cell lysate on the peptide functionalized FM sensor surface, no response was observed (Supplementary Figure 7 in S1 File). The RKO cell line was selected since it was showing the lowest basal β-catenin level compared to other cell lines (Supplementary Method 1; Supplementary Figure 8 in S1 File).

For FM, the normalized equilibrium response (R_eq_) obtained from each injection of protein or peptide in buffer and cell lysate is shown across the 18 molograms for a representative sensor is shown (Fig 4). These values were plotted against the injected concentrations of protein or peptide respectively, in order to compare the sigmoidal curve shape of SPR and FM (Fig 4). A clear similarity in the sigmoidal curves between SPR and FM experiments could be observed, indicating that the binding mode in both measurement systems were comparable. However, the Hill slope of the dose-response observed in FM was higher (1.88 ± 0.07, for 3 Hill slopes) in comparison to SPR (1.21, only one Hill slope) for experiments conducted in buffer (Fig 3A). This may indicate a higher surface density of ligands available for binding [50] in FM compared to SPR. Moreover, the planar nature of the FM surface contrasts with the three-dimensional dextran matrix used in SPR, which can significantly affect ligand orientation and accessibility. The dextran matrix may create steric hindrance or alter ligand orientation, influencing binding dynamics and reducing apparent ligand density and accessibility, thereby affecting the Hill slope observed in SPR [51–54]. This phenomenon aligns with findings from molecular dynamics simulations, which have shown that ligand orientation and conformational changes can significantly influence receptor activity and binding affinity [51,55]. Additionally, the SPR system might exhibit avidity effects due to multivalent interactions facilitated by the dextran matrix, contributing to lower observed Hill slopes. A modeling effort considering these interactions could provide a more comprehensive understanding of surface architecture impacts on binding [51,56]. While a higher Hill slope in FM indicates increased ligand density, other factors such as cooperative binding and conformational changes should also be considered. Cooperative binding could increase the Hill slope in FM if binding of one ligand increases the affinity for subsequent ligand binding. Similarly, conformational changes upon ligand binding may alter effective ligand density. This is supported by studies showing structural changes upon binding, which can alter ligand accessibility and binding site presentation [51,53]. The effect decreased when comparing the curves of SPR (1.21) in buffer and FM (1.6 ± 0.06) in cell lysate (Fig 3B). That could be explained by the presence of cell lysate components partially blocking the FM sensor surface and changing the conformation and the mode of action between the protein and ligand, thus decreasing the Hill slope. Such conformational changes are known to affect binding dynamics, as indicated by several studies [51–55]. The specificity of the β-catenin/TCF4(7–30) interaction was demonstrated in the competition experiments. The results underlined the outstanding performance of FM to detect specific interaction in a complex environment. The sigmoidal curve response plots for all sensors can be found in Supplementary Figures 4 and 5 in S1 File.

**Fig 5:**
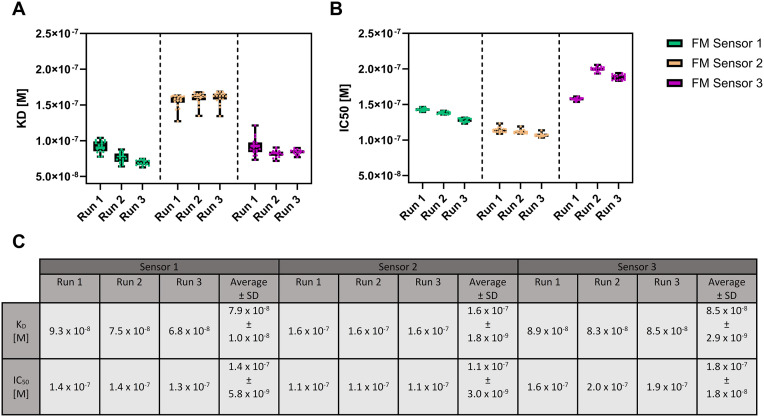
Overview of binding affinities (K_D_) and half maximum inhibitory concentrations (IC_50_) obtained in FM measurements performed in buffer. The experiments were in triplicates on 3 different sensors. The boxplot analysis represents the indivudual binding affinities (A, K_D_) and half maximum inhibitory concentrations (B, IC_50_) calculated from 18 indivudual molograms with software provided by Lino Biotec. The box contains 50% of the values and the median value is represented as line, while the whiskers contain the upper and lower 25% of all molograms of an experiment. (C) Median K_D_ and IC_50_ values calculated from 18 molograms as well as the avarage values calculated from three experiments with standart deviation.

**Fig 6:**
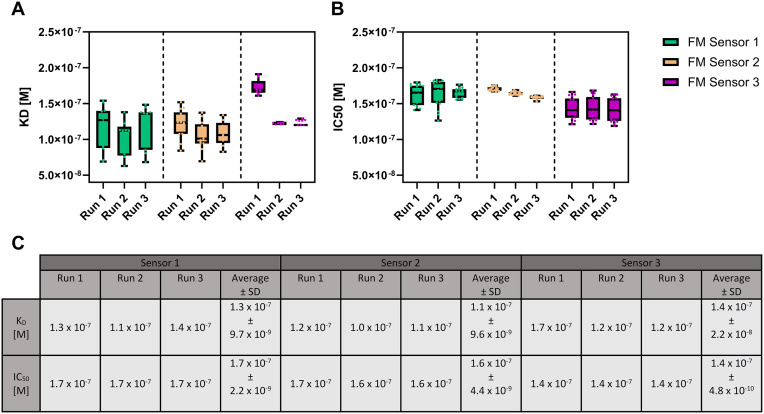
Overview of binding affinities (K_D_) and half maximum inhibitory concentrations (IC_50_) obtained in FM measurements performed in cell lysate (1 Mio cells/mL). The experiments were in triplicates on 3 different sensors. The boxplot analysis represents the indivudual binding affinities (A, K_D_) and half maximum inhibitory concentrations (B, IC_50_) calculated from 18 indivudual molograms with software provided by Lino Biotec. The box contains 50% of the values and the median value is represented as line, while the whiskers contain the upper and lower 25% of all molograms of an experiment. (C) Median K_D_ and IC_50_ values calculated from 18 molograms as well as the avarage values calculated from three experiments with standart deviation.

In order to evaluate the reproducibility of the binding affinity and half maximum inhibition constants determined in FM experiments, each of the 18 molograms on each chip in each experiment (run) were analyzed and the results were visualized with boxplots (Figs 5–6).

The K_D_ values determined in buffer from FM experiments ranged from 68 to 160 nM, while SPR analysis yielded values from 96 to 121 nM, demonstrating good comparability between both assays. Furthermore, the FM competition experiments with the free peptide resulted in IC_50_ values ranging from 110 to 200 nM whereas SPR yielded values ranging from 95 to 115 nM further confirming reproducibility and reliability of the FM measurements.

This consistency was observed not only across different molograms in the same experiment but also across multiple experiments on the same sensor as well as across different sensors. However, the remaining deviations could be attributed to differences in flow profiles within the FM measurement chamber. The FM measurement chamber is significantly lager, especially in the direction perpendicular to the flow (12.0 x 1.9 x 0.1 mm) compared to SPR (2.1 x 0.5x 0.05 mm) [57]. Consequently, when samples were injected into the FM chamber, border effects may have impacted the distribution and velocity of the samples across individual molograms. This could be resolved in the future by miniaturizing the sample chamber of FM. Moreover, although similar immobilization densities while functionalizing the FM sensors with the peptides were targeted, differences could be due to variations in the density at the level of chemical linkers (MTz and Tz) during the chip fabrication process. These differences can influence the final ligand densities immobilized on the sensor surface. This is not only true for the different sensors, but also for the different molograms on the same sensors, as the quality of the molograms varies due to reactive immersion lithography process that was not performed in a displacement Talbot mode for the chips used in this study [58]. Nonetheless, these effects were only insignificantly influencing the observed binding affinities and half-inhibition concentrations (K_D_ and IC_50_ values).

Next, the comparability of the binding data for the protein-peptide interaction were analyzed in cell lysate (Fig 6).

The determined K_D_ values for the protein-peptide interaction in cell lysate using FM ranged from 100 to 140 nM, while SPR analysis yielded 96–121 nM for the same interaction in buffer. These results demonstrate that FM can reliably measure this interaction in a crude sample, in presence of non-specific binders found in cell lysate on the chip matrix. This assay would not be straightforward to implement with SPR, highlighting the power of FM’s background subtraction, without intensive and time-consuming assay optimization. Furthermore, FM experiments in cell lysate revealed an IC_50_ between 140–170 nM in excellent agreement with the results obtained using SPR in buffer (95–115 nM). Also, this assay would involve considerable optimization on an SPR device. In summary, FM enabled the comparable and reproducible measurement of binding affinities and half maximum inhibition concentrations for protein-peptide interactions in buffer and cell lysate whereas SPR was limited to measurements in buffer.

Slight deviations of binding affinities and inhibition concentrations between individual molograms on the same sensor (within a factor of 2) could again be attributed to variations in the flow profile within the measurement chamber. Likely, not all molograms received the exact same concentration of samples due to border effects in the measurement chamber, hence small variations in the calculated K_D_ and IC_50_ values were expected and observed. Furthermore, initial coupling of the incident light beam to the sensor chip (waveguide) is different when changing the sensors or experiments [27]. Important in this case would be to ensure similar initial coupling conditions when comparing binding results. Additionally, a slight decrease in binding affinity (higher K_D_ values) in cell lysate was observed compared to the experiments performed in buffer. This observation could be explained by β-catenin or free TCF4(7–51) peptide interacting with components of the cell lysate, leading to competing interactions and lowered effective concentrations.

One key finding of our study was that molecules randomly adsorbed from the cell lysate onto the FM sensor surface did not contribute to the recorded signal during the experiments. This highlights a significant advantage of FM diffractive sensor over classical refractometric sensors like SPR, where randomly adsorbed molecules cause a refractive index change, leading to a signal increase that can obscure the specific binding signal of the analyte to the ligand, even after subtraction of the signal measured in the reference flow channel.

In contrast, FM effectively eliminates this issue. On a backfilled FM sensor, randomly adsorbed molecules distribute equally between ridges and grooves due to the highly similar chemical environments and the sub-micron proximity of reference and signal regions. Since the FM signal only increases when mass accumulates unequally between ridges and grooves, no signal is generated from non-specific binding. Specific interactions, such as the binding of β-catenin to the peptide on the sensor surface, result in an unequal distribution of mass between ridges and grooves, thereby producing a measurable signal only upon specific binding.

## Conclusion

The presented study validated that focal molography, a novel label-free biosensor, reproducibly determined the binding affinities of a protein to a peptide. The measured affinity constants were compared to those obtained using the gold standard technology – surface plasmon resonance. We demonstrated that the affinity constants determined by FM are consistent with those measured by SPR. Additionally, we successfully measured these constants not only in buffer but also in a complex medium, specifically cell lysate, in a reproducible manner. While significant non-specific adsorption of biomolecules from the cell lysate to the sensor surface was occurring, our results show that these non-specific interactions did not contribute to the recorded signal in focal molography. This is in a strong contrast to refractometric sensors like SPR, where non-specific interactions obscured meaningful measurements of affinity constants. Focal molography thus emerges as a promising new technology enabling biomolecular interaction studies in real biological samples, which is crucial for developing drug candidates and new diagnostic tools. Future studies and technology advancements focus on exploring and enhancing the sensitivity limits of this technology and its throughput. While this study investigated relatively large biomolecules such as proteins and peptides, small molecules will present new challenges that need to be addressed. The device used in this study was a prototype that is still undergoing development and refinement. Now that the feasibility of the measurements was demonstrated, optimizing the hardware will be key to improving the overall system performance. Nonetheless, the results presented here demonstrate that focal molography is a new and promising method in the field of biomolecular interaction analysis, with significant potential for advancing research and applications in this area.

## Supporting information

S1 File Supporting Figures and Methods. (PDF)

S1 Data Raw Data. (XLSX)
