# Supplementary material for: Exploring β-catenin and TCF4 interaction in complex environments by means of novel biosensing platform focal molography
Source: PLoS One. 2025 Sep 30;20(9):e0333554. doi: 10.1371/journal.pone.0333554 (PMC12483217; doi:10.1371/journal.pone.0333554)
Supplement: S1 File — (PDF) [file pone.0333554.s001.pdf]

Supplementary Information

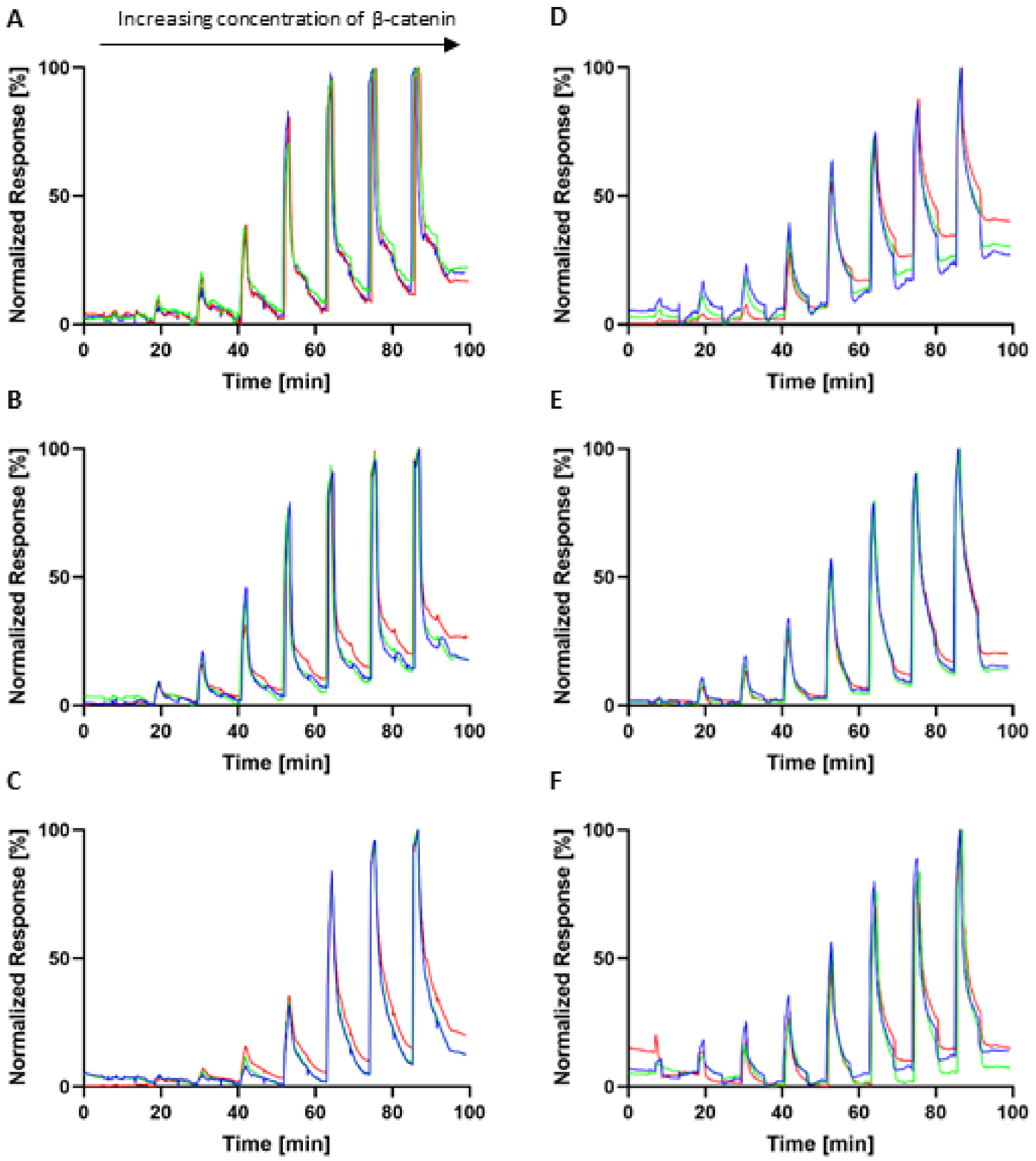

SI Figure 1: Time traces collected by titration of β-catenin up to 1 µM over TCF4(7-30) sensor surface to determine binding affinity constant (K_D). **A,B** and **C** show the time traces from experiments in buffer conducted on three individual sensors. The normalized (highest signal = 100%, lowest signal = 0%) median mologram (from 18 molograms per experiment) from three experiments are superimposed. The 3 individual time traces collected on each sensor are shwon in green, red and blue. **D, E** and **F** show time traces from analouge experiments performed in cell lysate (1 Mio cells / mL).

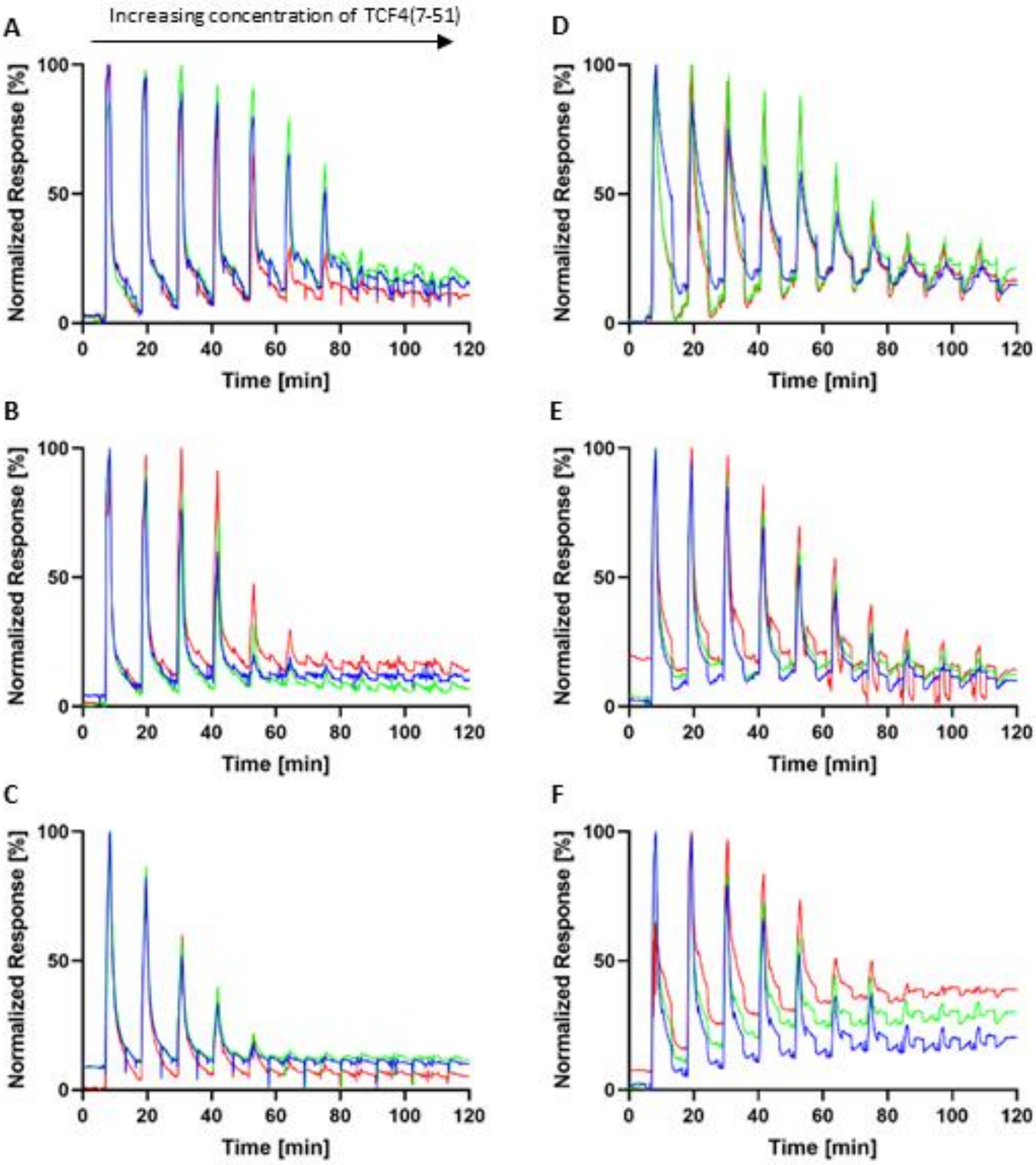

SI Figure 2: Time traces collected by titration of TCF4(7-51) up to 1 µM in complex with 250 nM β-catenin over TCF4(7-30) sensor surface to determine half maximum inhibitory constant (IC_{50}). **A,B** and **C** show the time traces from experiments in buffer conducted on three individual sensors. The normalized (highest signal = 100%, lowest signal = 0%) median mologram (from 18 molograms per experiment) from three experiments are superimposed. The 3 individual time traces collected on each sensor are shwon in green, red and blue. **D, E** and **F** show time traces from analouge experiments performed in cell lysate (1 Mio cells / mL).

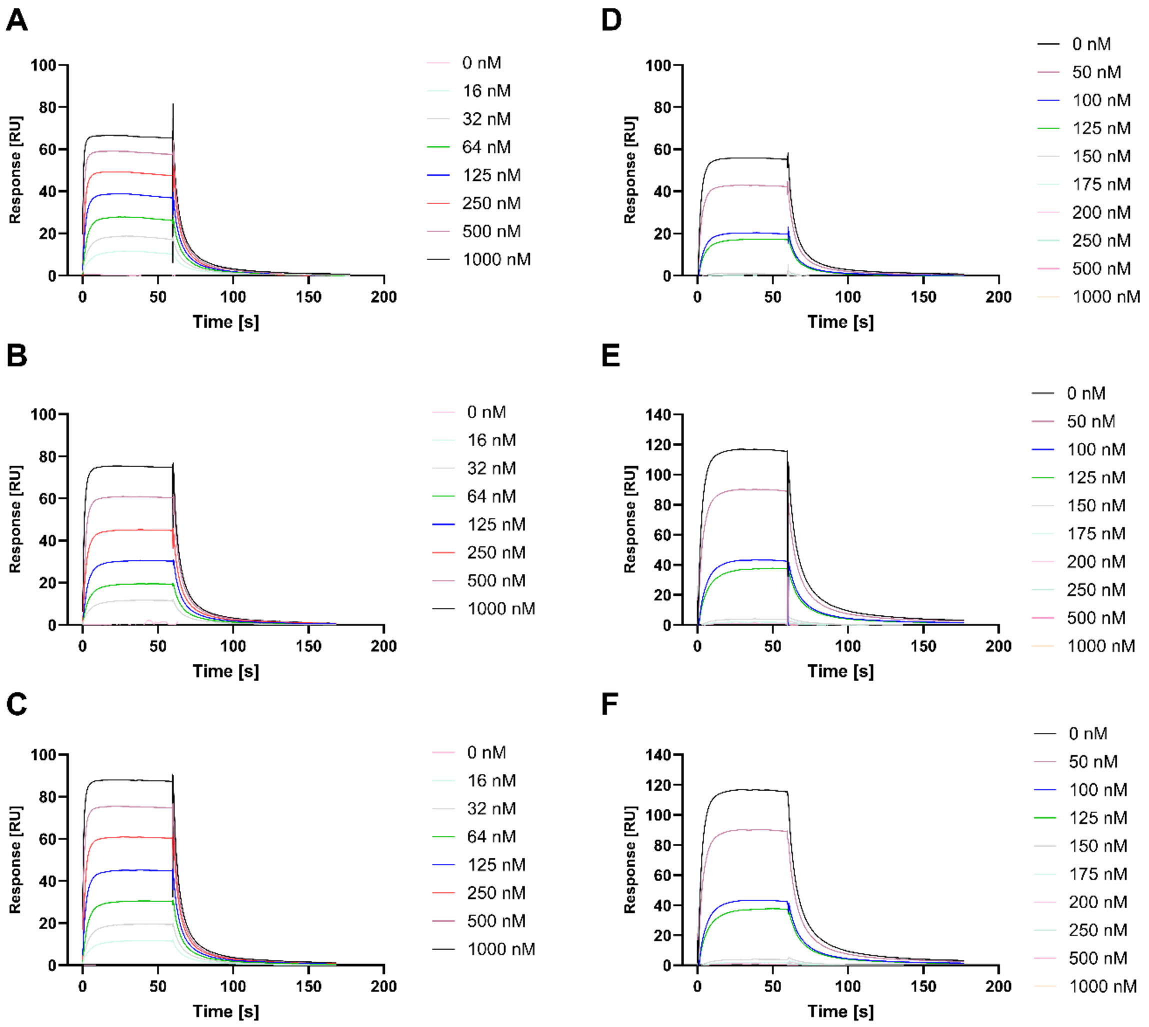

SI Figure 3: SPR-based binding and competition experiments with β-catenin and immobilized TCF4(7-30). **A**, **B** and **C:** Dose-response binding curves monitored by β-catenin titration on TCF4(7-30) surface to measure K_D in buffer with concentration of β-catenin in the legend. **D**, **E** and **F:** Dose-response binding curves monitored by TCF4(7-51) titration to 250 nM β-catenin in solution on TCF4(7-30) surface to measure IC_{50} in buffer with concentration of TCF4(7-51) in the legend. In **A** and **D**, the curves of the channel with 5 RU immoblization density of TCF4(7-30) is shown. In **B** and **E**, the curves of the channel with 25 RU immoblization density of TCF4(7-30) and in **C** and **F**, the curves of the channel with 50 RU immoblization density of TCF4(7-30).

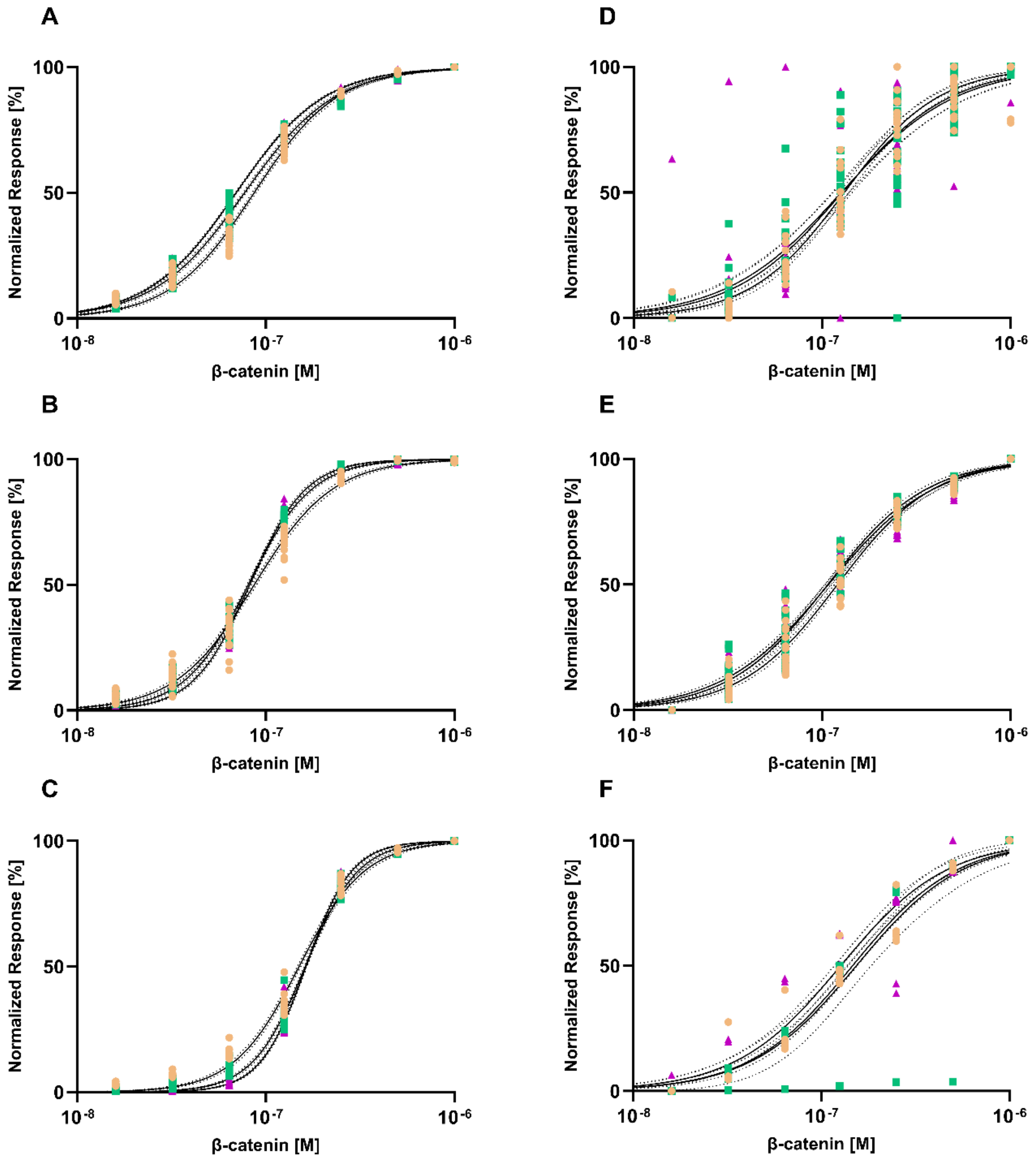

SI Figure 4: Sigmoidal dose response curves for β-catenin/TCF4(7-30) interaction monitored in FM measurements in three experiments on 18 molograms. The amplitude of the FM signal (response) is normalized (y-axis) to 100% and plotted versus concentration of β-catenin (x-axis) in buffer (**A**, **B**, **C**) and cell lysate (**D**, **E**, **F**) in triplicates (three different colors). In all curves obtained from three experiments the 95% confidence interval band is plotted as dotted line. The sigmoidal dose response curves were fitted with the non-linear regression model in Graphpad Prism 10.4.1 (log(inhibitor) vs. normalized response – Variable slope).

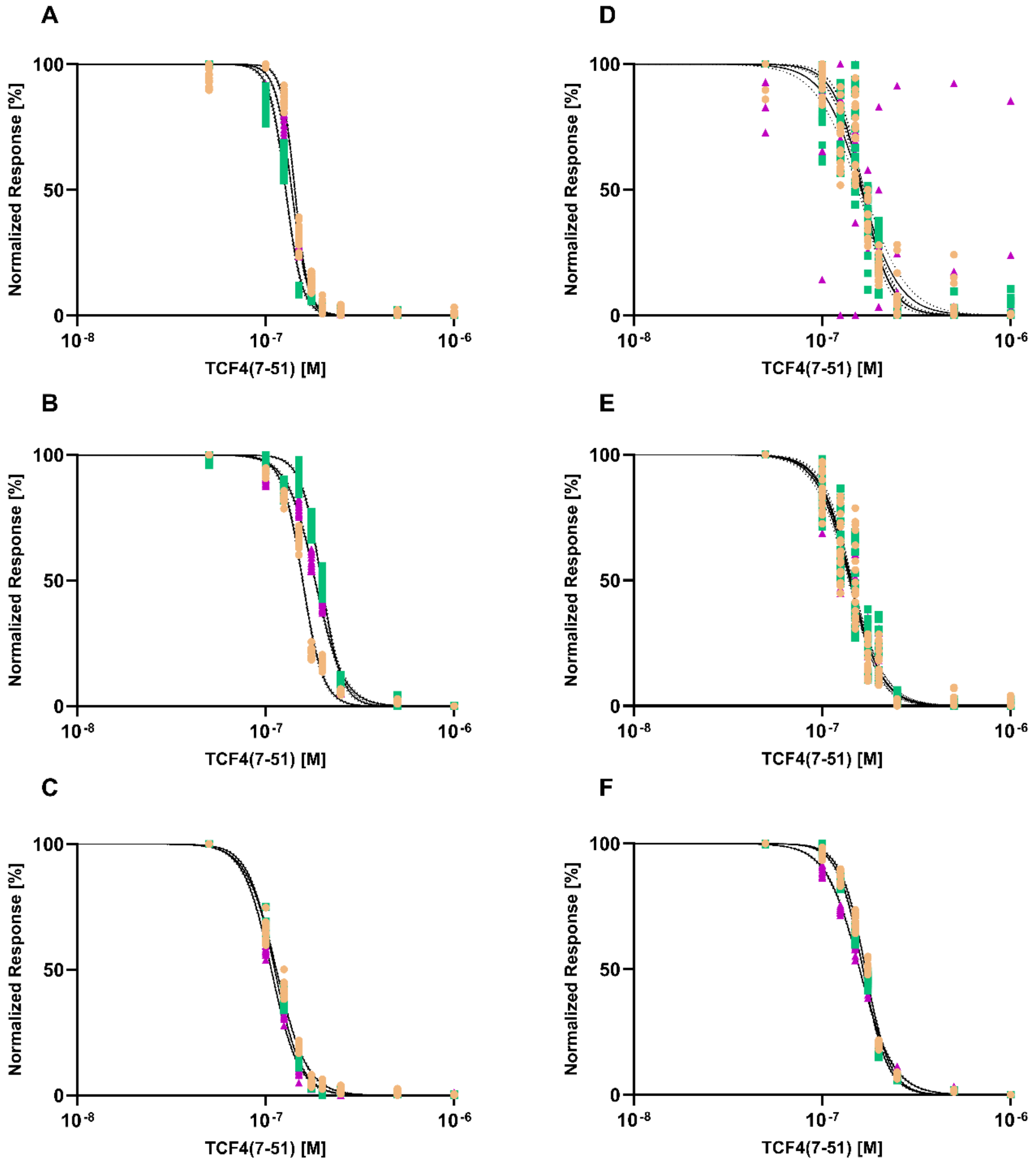

SI Figure 5: Sigmoidal dose response curves for β-catenin/TCF4(7-51) competition monitored in FM measurements in three experiments on 18 mologram. The amplitude of the FM signal (response) is normalized (y-axis) to 100% and plotted versus concentration of TCF4(7-51) (x-axis) in buffer (**A**, **B**, **C**) and cell lysate (**D**, **E**, **F**) in triplicates (three different colors). In all curves obtained from three experiments the 95% confidence interval band is plotted as dotted line. The sigmoidal dose response curves were fitted with the non-linear regression model in Graphpad Prism 10.4.1 (log(inhibitor) vs. normalized response – Variable slope).

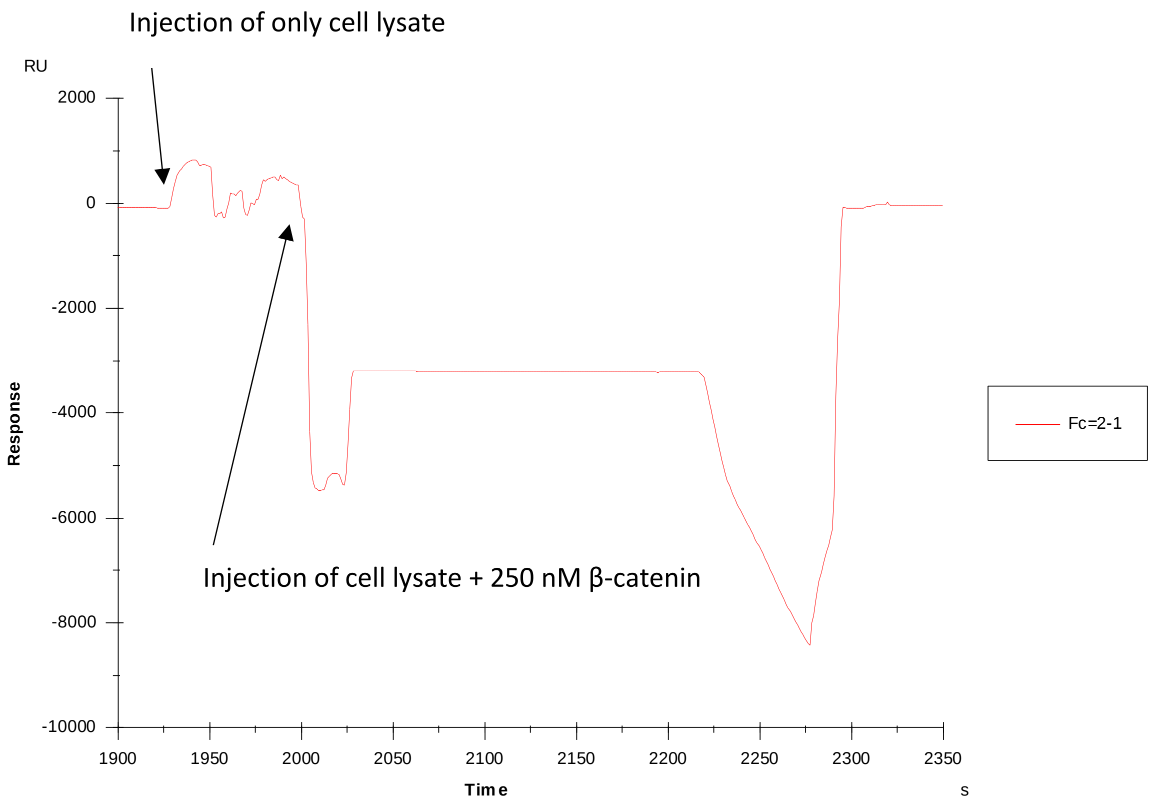

SI Figure 6: SPR raw data of manual injection of cell lysate and β-catenin injection into SPR system (flow channel 2-1). The same cell lysate (1 Mio cells/mL) that had nearly no response in FM, lead to massive response when injected into the SPR system.

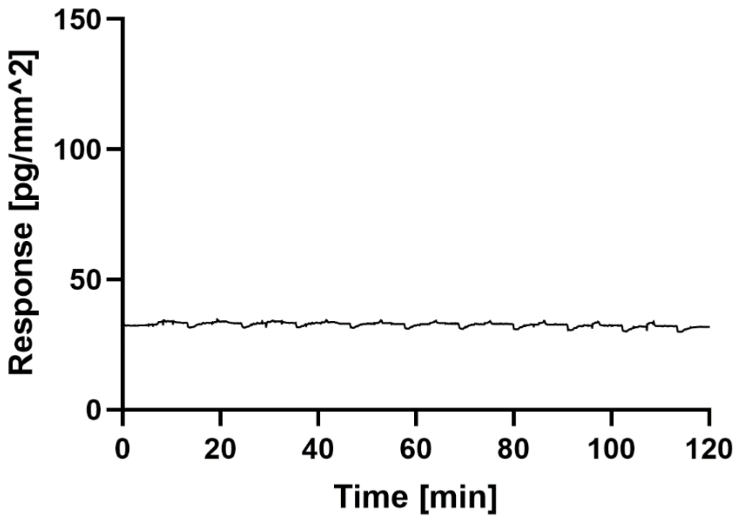

SI Figure 7: Cell lysate injections over TCF4(7-30) peptide surface: Control experiment to address unknown or unexpected specific interactions from the cell lysate with the peptides immobilized on the FM sensor surface monitored in 10 consecutive injections of cell lysate.

Supplementary method 1: Cell culture and western blot analysis

RKO, Colo320DM, HEK293 cells were obtained from the American Type Culture Collection. RKO and HEK293 were cultured in DMEM, Colo320DM in RPMI ATCC medium, all medium were supplemented with 10% fetal bovine serum and 100 µg/ml penicillin-streptomycin (all from Gibco). All lines were maintained under humidified atmosphere containing 5% CO_2 at 37°C. Cells were seeded in triplicates and treated for 24 hours with DMSO or with the GSK3 inhibitor Laduviglusib (CHIR-99021, from Selleckchem). Cells were lysed using RIPA buffer (Sigma) supplemented with Halt Protease and Phosphatase Inhibitor Cocktail (ThermoFisher Scientific) and Benzonase nuclease (Sigma). The protein samples were quantified using Pierce™ BCA Protein Assay (ThermoFisher). Samples were analyzed with a JESS capillary western blot system (ProteinSimple, Bio-Techne) following the manufacturer's protocol and the default settings. Protein separation was done with a 12-230kDa separation module (ProteinSimple). Protein detection was done with anti β-Catenin (6B3) primary antibody, anti GAPDH primary antibody (both from Cell Signaling Technology) and Anti-Rabbit detection module (ProteinSimple). Results were visualized and analyzed with the Compass for SW software (ProteinSimple, Version 6.2.0). Graphpad Prism was used to generate the histogram and perform the statistical analysis.

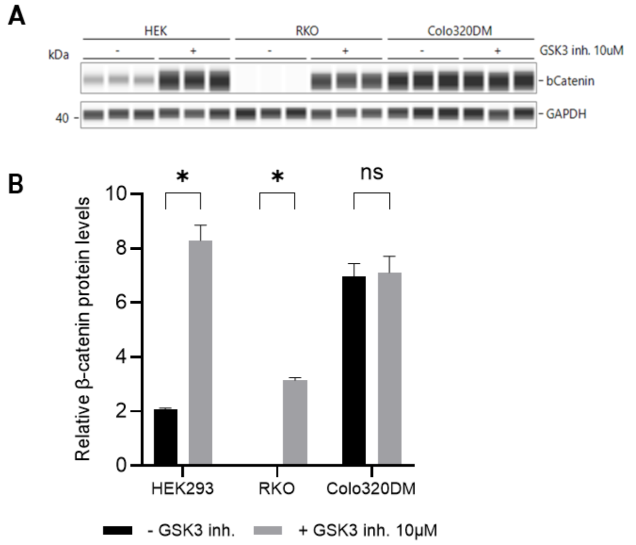

SI Figure 8: Beta-catenin protein levels measured in various cells lines. **A:** capillary Western Blot analysis. **B:** relative beta-catenin quantification, the graph shows the mean of the 3 replicates ± SEM, statistical significance was determined by an unpaired Student's t-test (∗: $p < 0.05$, ns: not significant).

A

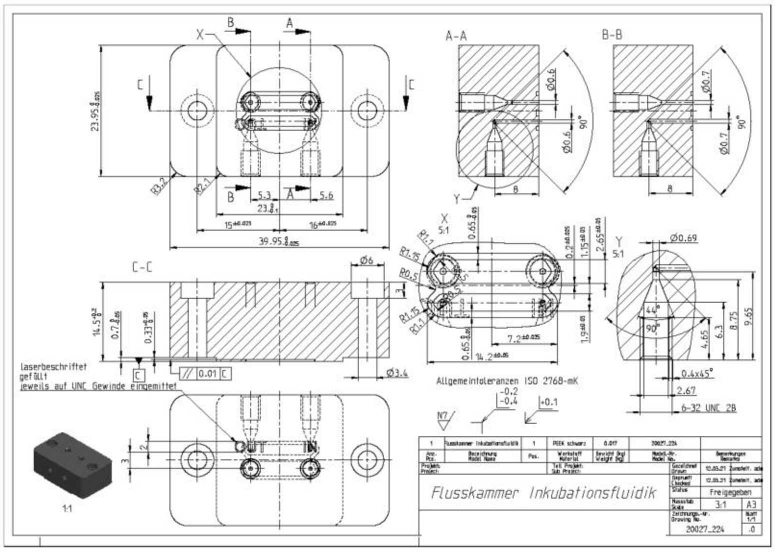

B

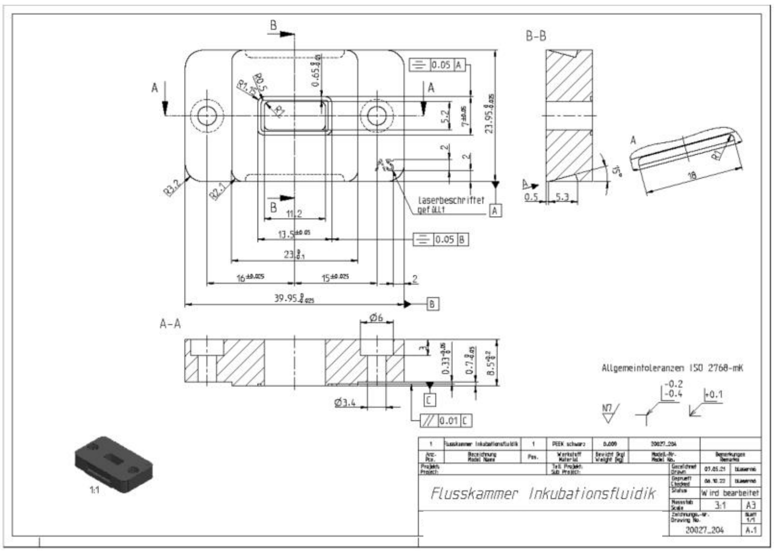

C

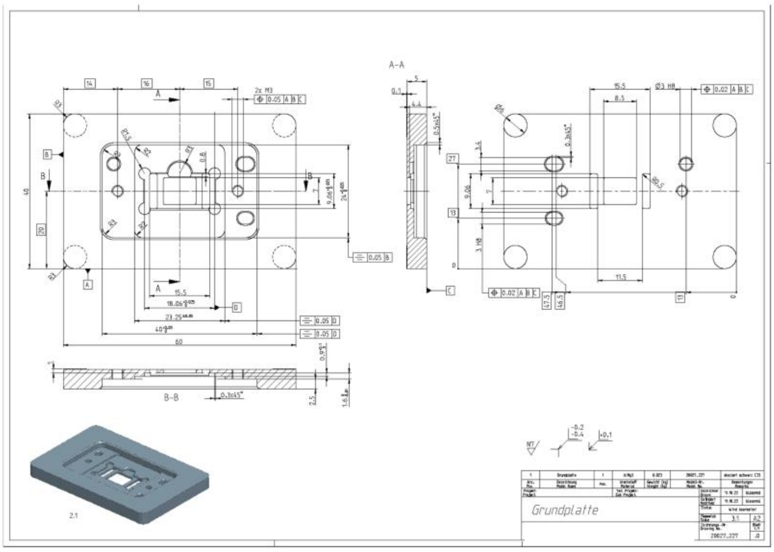

SI Figure 9: Flow chamber design for FM. **A**: Double flow chamber used for affinity measurements. **B**: Open chamber for peptide immobilization on FM sensor surface. **C:** Baseplate, where the sensor chip is placed and the flow chamber mounted on top. The baseplate was used in combination with **A** or **B**.

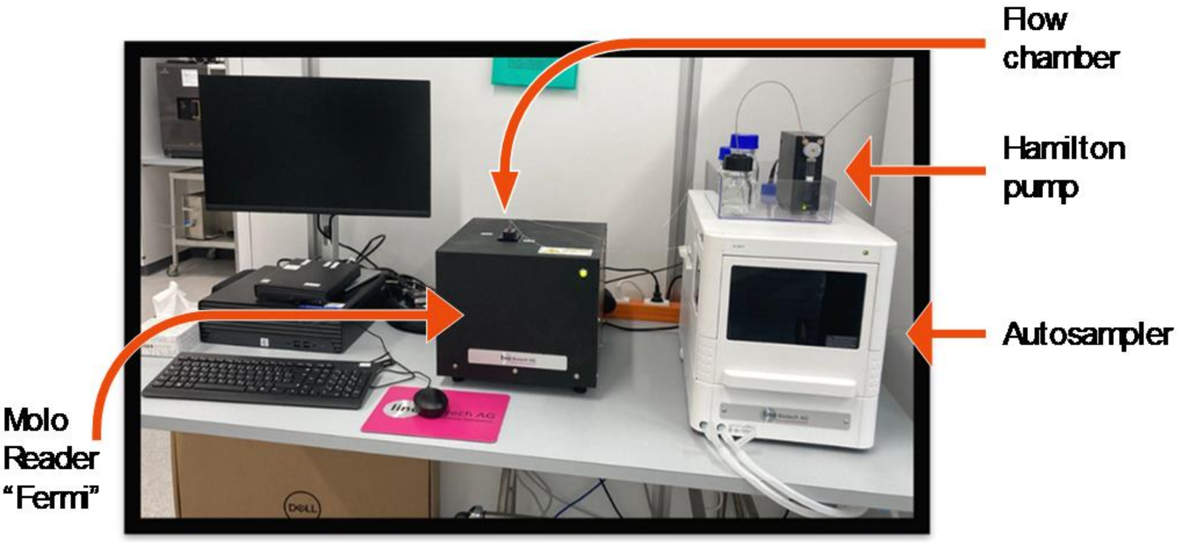

SI Figure 10: FM reader setup: The "Molo Reader, Generation Fermi" was used to measure the intensity of the mologram on the sensor, which was mounted into the "Flow Chamber". The samples were delivered from the "Autosampler" to the flow chamber. Buffer and samples were transported through the tubing via the "Hamilton Pump".
